# Humoral epitope dominance and immune imprinting by SARS‐CoV‐1 and SARS‐CoV‐2 vaccines

**DOI:** 10.1111/imcb.70072

**Published:** 2026-01-06

**Authors:** Deborah L Burnett, Ania Moxon, Anupriya Aggarwal, Katherine JL Jackson, Catherine Cotter, Anouschka Akerman, Amanda Russell, Rachel Kalman, David Langley, Jake Y Henry, Daniel Christ, Rowena A Bull, Robert Brink, Anthony D Kelleher, Hans‐Martin Jäck, Stuart Turville, Bernard Moss, Christopher C Goodnow

**Affiliations:** ^1^ School of Biomedical Sciences, Faculty of Medicine and Health UNSW Sydney Sydney NSW Australia; ^2^ Garvan Institute of Medical Research Sydney NSW Australia; ^3^ Kirby Institute UNSW Sydney NSW Australia; ^4^ Cellular Genomics Futures Institute UNSW Sydney Sydney NSW Australia; ^5^ Laboratory of Viral Diseases National Institute of Allergy and Infectious Diseases, NIH Bethesda MD USA; ^6^ Institute for Molecular Bioscience University of Queensland St Lucia QLD Australia; ^7^ University of Notre Dame Notre Dame NSW Australia; ^8^ School of Clinical Medicine, Faculty of Medicine and Health UNSW Sydney Sydney NSW Australia; ^9^ Division of Molecular Immunology, University Hospital Erlangen University of Erlangen‐Nürnberg Erlangen Germany

**Keywords:** Epitope dominance, humoral immunity, modified vaccinia ankara, SARS‐CoV‐1, SARS‐CoV‐2, vaccines

## Abstract

Long‐lasting protective immunity against sarbecoviruses is hampered by the dominance of elicited antibodies to variable parts of the Spike protein, allowing ongoing viral escape and evolution. We investigated Modified Vaccinia Ankara (MVA) vaccine candidates expressing the SARS‐CoV‐1 or SARS‐CoV‐2 Spike for their ability to induce antibodies targeting different epitopes on the SARS‐CoV‐2 Receptor Binding Domain (RBD), including those with wide variant conservation. We also explored the capacity of these different Spike proteins to induce broad cross‐reactive or cross‐neutralizing B cells against multiple variants. This revealed that the SARS‐CoV‐1 Spike induced distinct patterns of epitope dominance compared to the traditional SARS‐CoV‐2 Spike antigens. Following immune imprinting by previous exposure to ancestral SARS‐CoV‐2 Spike, the epitope dominance patterns induced by SARS‐CoV‐1 and SARS‐CoV‐2 vaccines still differed, with most of the germinal center response consisting of *de novo* recruited B cells. In addition to the *de novo* response, B cells with germline cross‐reactivity to both antigens further increased their binding toward the most recently immunized antigen. Interestingly, we found that, while SARS‐CoV‐2 vaccinated animals were extremely capable of mounting an antigen‐specific germinal center and plasmablast response to a booster immunization with SARS‐CoV‐1, SARS‐CoV‐2 boosters were less capable of inducing SARS‐CoV‐2 specific B cells following prior SARS‐CoV‐1 vaccination. These findings have broad implications for the implementation of vaccine strategies against emerging coronavirus variants and potential future coronavirus spillover events. The implications stemming from a fundamental directionality of immune imprinting and epitope dominance may have wider implications for noncoronavirus antigens.

## Graphical abstract

We investigated Modified Vaccinia Ankara (MVA) vaccine candidates expressing the SARS‐CoV‐1 or SARS‐CoV‐2 Spike, as a primary or booster immunization, for their ability to induce antibodies targeting different epitopes on the SARS‐CoV‐2 RBD and broad cross‐reactive or cross‐neutralizing responses in C57BL/6 and antibody humanized (Ig‐humanized) mice.

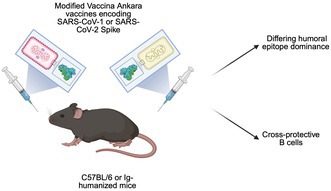

## INTRODUCTION

Although SARS‐CoV‐2 (CoV‐2) has become an endemic human pathogen, infections continue to result in serious health consequences including hospitalizations and death. In particular, the burden of disease among immunocompromised individuals remains extremely high.^1^ Current COVID‐19 vaccines offer only transient capacity to limit viral load or transmission with later CoV‐2 variants, further highlighting the ongoing risk to vulnerable populations.^2^, ^3^ The risk of future zoonotic spillover and/or viral recombination events highlights the ongoing need for exploration into vaccine strategies that target broadly conserved epitopes shared across the sarbecovirus families.^4^, ^5^

SARS‐CoV‐1 (CoV‐1) may hold advantages as a vaccine antigen compared to traditional CoV‐2 vaccine approaches. CoV‐1 convalescent patients vaccinated with COVID‐19 vaccines show greater cross‐reactivity than CoV‐2 convalescent patients immunized with the same vaccines.^6^ Many next generation vaccine strategies are exploring CoV‐1 as a potential vaccine antigen, either as a booster vaccine,^7^, ^8^ or as an element of a combined nanoparticle^9^, ^10^, ^11^ or mRNA^12^ cocktail vaccine. Given this increasing focus on CoV‐1 as a vaccine antigen, there is a strong need for studies exploring the differences in humoral epitope dominance between the CoV‐2 and CoV‐1 Spike. Exploration of humoral epitope dominance for CoV‐2 has traditionally only been focused on the serum antibody repertoire.^10^ However, this strategy only accounts for pooled polyclonal antibodies and does not allow for assessment at the single cell level or for the development and maturation of specificity to each antigen. Exploring the cross‐reactivity and epitope dominance of individual B cells would allow us to highlight the capacity of CoV‐1 vaccines to induce B cells targeting conserved sites and the potential of this strategy as a booster immunization.

Neutralizing antibodies against CoV‐2 have been categorized as canonically recognizing five distinct patches on the Receptor Binding Domain (RBD).^13^, ^14^ Most antibodies induced by natural infection or vaccination with the CoV‐2 Spike protein RBD are Class 1/2, which bind to the poorly conserved ACE2 Receptor Binding Surface (RBS).^15^, ^16^, ^17^, ^18^ Several more highly conserved RBD epitopes have been explored for their capacity to induce cross‐protective immunity. The Class 3 epitope, as represented by the canonical S309 antibody,^19^ represents a conserved buried epitope only available in the up RBD conformation.^13^ The Class 4 site was originally exemplified by the CoV‐1 derived antibody CR3022; however, more potent CoV‐2 neutralizing Class 4 antibodies have since been described.^15^, ^20^ The Class 5 epitope was more recently discovered, epitomized by the canonical S2H97 antibody.^14^

At the serum level in humans, it has been confirmed that vaccination can alter epitope immunodominance profiles, with the Beta variant leading to a higher response toward the Class 3, rather than Class 1/2 epitopes.^21^ This, as proof of principle highlighted the importance of antigenic selection in vaccine design to optimize the generation of cross‐protective immune responses. We have previously shown in C57BL/6 mouse models that CoV‐1 immunization directed the humoral immune response toward the conserved Class 4 epitope of the sarbecovirus RBD.^15^ Although this finding was informative, the artificial nature of the antigen exposure in that study, where antigens were conjugated to foreign erythrocytes, opened justifiable questions as to the translatability and applicability of those findings in the context of COVID‐19 vaccines. Furthermore, the emergence of Omicron variants, which evade neutralization by previously cross‐neutralizing antibodies targeting the Class 4 epitope,^22^ highlighted that explorations of humoral epitope dominance between CoV‐1 and CoV‐2 need to extend beyond just the Class 4 epitope and into other conserved epitopes that better maintain potency against Omicron variants.^14^, ^19^

To better explore the humoral epitope dominance of CoV‐1 and CoV‐2 Spike, we utilized the Modified Vaccinia Ankara (MVA) vaccine system as a robustly tested and physiologically relevant vaccination model system. Developed as a safer alternative, replication‐incompetent variant of the vaccinia vaccine, MVA vaccines have been extensively explored in widescale population studies and have been shown to be safe even in immunosuppressed individuals.^23^ Although MVA vaccines are less potent than their unattenuated relatives, potency issues can be overcome through the route of administration.^24^, ^25^, ^26^ MVA vaccines have proven critical in the global effort to protect against mpox, receiving therapeutic approval and being utilized extensively in multiple countries worldwide on a population scale.^27^, ^28^ CoV‐2 MVA vaccines have been developed and explored by several groups which have confirmed their capacity to generate robust and protective immunity in both mouse and hamster models.^23^, ^24^, ^26^, ^27^, ^29^, ^30^, ^31^, ^32^, ^33^, ^34^, ^35^, ^36^ Immunization with prefusion stabilized CoV‐1 Spike MVA as a primary immunization was able to induce low titer cross‐protective antibodies binding Ancestral CoV‐2 despite its distant sequence homology.^31^ Evidence exploring protection against mismatched MVAs has revealed that immunization with Ancestral CoV‐2 Spike induced low cross‐neutralizing antibodies against later Omicron variants. However, this still translated into protective immunity in challenge models.^30^ Immunization with Omicron Spike induced minimal neutralizing antibodies cross‐reacting to CoV‐2 Ancestral; however, it still showed some capacity to reduce the titer of infection with CoV‐2 Ancestral.^30^ Given that the majority of the population has been exposed to CoV‐2 through previous vaccination and infection, any vaccination strategy seeking to direct the immune response toward conserved antibody targets needs to account for the additional challenge of overcoming the bias caused by immune imprinting/original antigenic sin toward the initially exposed targets.^37^, ^38^, ^39^, ^40^ At the serum level, it has been demonstrated that vaccination with an MVA Omicron booster following primary MVA CoV‐2 Ancestral immunization can result in an increased neutralizing antibody titer against both strains.^30^ This is consistent with evidence in humans which has shown that Omicron bivalent boosters and breakthrough infection can expand both *de novo* and cross‐reactive RBD‐specific memory B cells.^38^, ^41^, ^42^, ^43^, ^44^, ^45^

Here, we have extended the analysis of immune imprinting to explore the potential for directing the antibody response to conserved sites of the CoV‐2 Spike at the cellular level within specific B‐cell compartments following CoV‐1 or CoV‐2 homologous or heterologous MVA booster immunizations.

## RESULTS

### Immunization with CoV‐1 or CoV‐2 MVA induces a serum response against sarbecovirus RBDs

To explore the humoral response to prefusion stabilized CoV‐1 and CoV‐2 Spike MVA vaccines,^31^ we vaccinated C57BL/6 mice with either 2 × 10^8^ PFU CoV‐1 or CoV‐2 Spike MVA on days 0 and 14 and analyzed the response on day 28.

**Figure 1:**
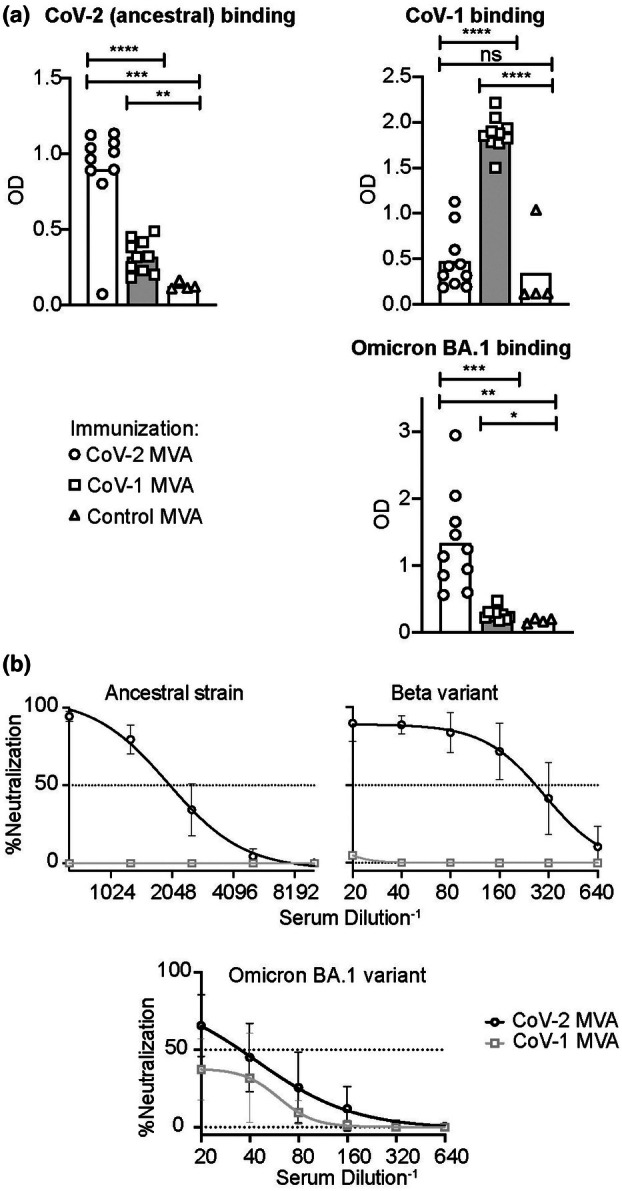
Comparison of the serum immune response from immunization of CoV‐1 or CoV‐2 Spike MVA. C57BL/6 mice were immunized with 2 × 10^8^ PFU IV of CoV‐1 MVA, CoV‐2 MVA, or control MVA on days 0 and 14 and the immune response examined on day 28. **(a)** Optical density (OD) of serum antibody binding to the indicated coronavirus receptor binding domains, measured by ELISA using a second‐stage antibody against kappa light chains. Data points represent individual mice. Columns denote arithmetic means. Statistics incorporated one‐way ANOVA. If ANOVA revealed significant differences unpaired *t*‐tests were applied to compare between different groups. Welch's correction was applied if variances between groups were statistically different. ns = not significant. *P < 0.05, **P < 0.01, ***P < 0.001, ****P < 0.0001. **(b)** Live virus neutralization of the indicated variants causing a cytopathic effect in HEK292T cells following incubation with serum at the indicated dilutions, from C57BL/6 mice vaccinated with CoV‐2 or CoV‐1 MVA. Error bars show mean ± SEM. IC50 was calculated by nonlinear regression (curve fit). *N* = 10 mice per group for CoV‐1 and CoV‐2 MVA and 4 mice for control MVA. Data pooled from two independent experiments.

Both CoV‐1 and CoV‐2 MVA vaccines were able to induce serum antibodies against their encoded target antigen measured by ELISA (Figure 1a). Both recombinant vaccines also induced a cross‐reactive serum antibody binding titer against the reciprocal antigen (i.e., CoV‐1 MVA against CoV‐2 and vice versa). However, consistent with other studies,^31^ this antibody titer was significantly lower than that against the target encoded in the immunized MVA vaccine. While both CoV‐2 and CoV‐1 MVA vaccines induced a serum antibody response against the Omicron BA.1 variant, this response was significantly higher following immunization with CoV‐2 MVA compared to CoV‐1. This is likely to reflect Omicron BA.1 Spike sharing 97% amino acid homology to the Ancestral CoV‐2 Spike utilized in the MVA design, compared to 75% homology against CoV‐1.

Serum from the immunized mice was also tested for live virus neutralization against the Ancestral CoV‐2, Beta and Omicron BA.1 variants using high‐content analysis of cytopathic effect in HEK292T cells (Figure 1b).^22^ Mice vaccinated with CoV‐2 MVA generated 220‐fold higher neutralizing antibody titers against the Ancestral virus (IC25: 3059 vs 14) than mice vaccinated with CoV‐1 MVA. This disparity was reduced in magnitude when serum neutralizing titer was compared against later variants. The Ancestral CoV‐2 MVA vaccinated mice had 88‐fold higher neutralizing antibodies against the Beta variant, which only contains 8 mutations of difference from the ancestral strain (IC25: 442 vs 5), and only 1.6‐fold, nonsignificant difference in neutralization of the Omicron BA.1 variant (IC25: CoV‐1:50, 95% CI 37–63; CoV‐2:81 95% CI 63–102).

### Immunization with CoV‐1 or CoV‐2 MVA vaccines to induce B cells binding sarbecovirus RBDs in a naïve murine and human Ig‐repertoire

The specificity of antibody responses to the recombinant MVA vaccines was measured at single‐cell resolution by staining with RBD tetramers from CoV‐2 (Ancestral and Omicron) and from CoV‐1 followed by flow cytometric analysis of GC cells (B220^+^, CD95^+^, CD38^−^, IgD^−^) and plasmablasts (PB, B220^+^, CD138^+^, IgD^−^) (Supplementary figure 1). The response was analyzed in C57BL/6 mice and in Ig‐humanized mice (Trianni mice), where human antibody V, D, and J elements replace the mouse elements at the heavy chain and kappa light chain loci.^15^, ^46^

**Figure 2:**
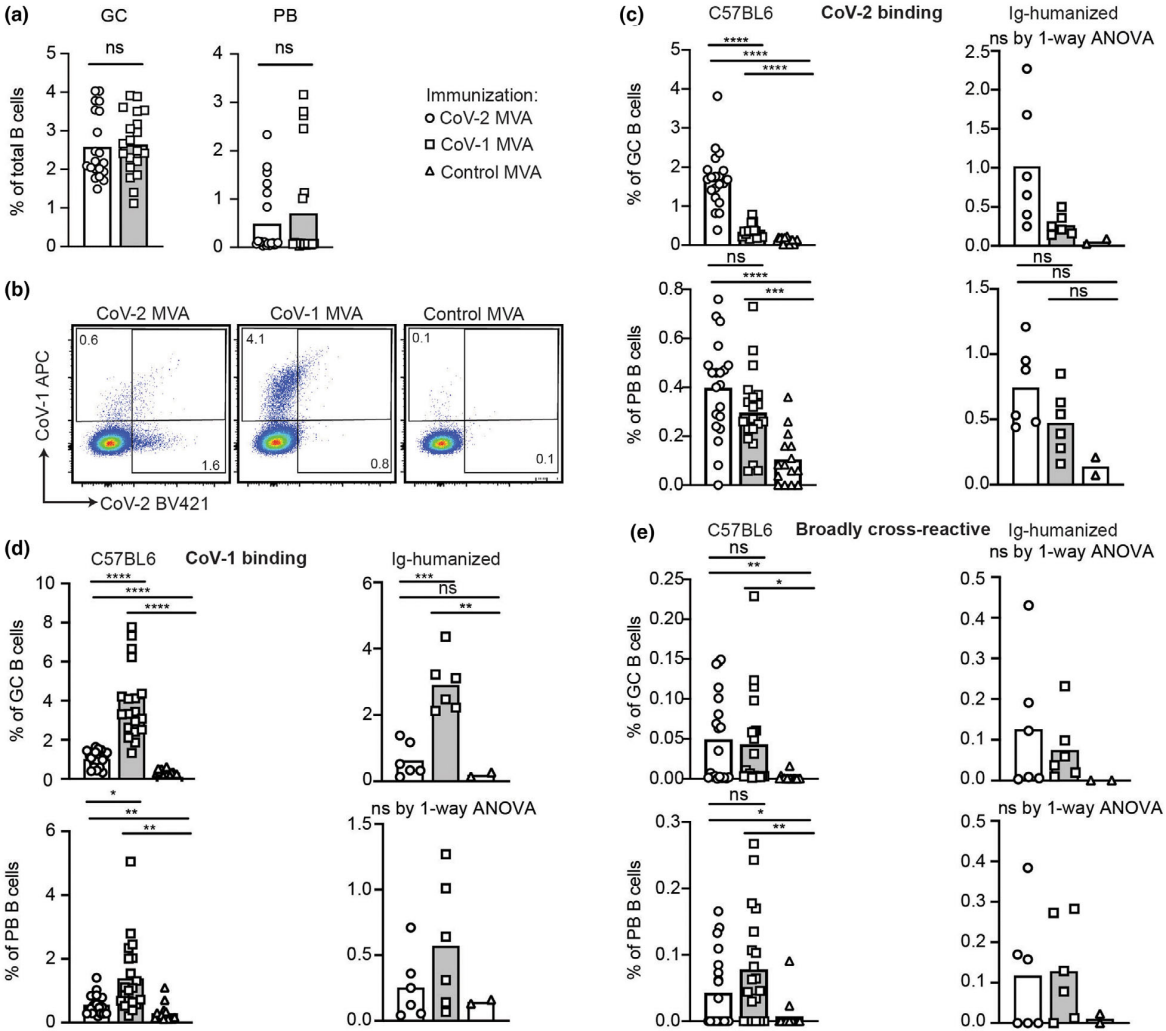
Single cell specificity of RBD antibody response in C57BL/6 and Ig‐humanized mice given CoV‐2 or CoV‐1 Spike recombinant MVA. C57BL/6 and Ig‐humanized C57BL/6 mice were immunized with 2 × 10^8^ PFU IV of CoV‐1, CoV‐2 or control MVA on days 0 and 14 and the immune response examined on day 28. **(a)** Total germinal center (GC) B cells (left) or plasmablasts (PB) (right) in the spleen of mice immunized with CoV‐1 or CoV‐2 MVA. **(b)** Representative flow cytometric plots of GC B cells showing binding to CoV‐1 and CoV‐2 (Ancestral) RBD tetramers. **(c–e)** Percentage of GC B cells or PB in individual C57BL/6 (left) or Ig‐humanized (right) mice binding to CoV‐2 RBD tetramers (c), CoV‐1 RBD tetramers (d), or CoV‐2 (Ancestral), CoV‐2 (Omicron BA.1), and CoV‐1 RBD tetramers concurrently (e). Data points represent individual mice; columns denote arithmetic means. Statistics incorporated one‐way ANOVA. If ANOVA revealed significant differences, unpaired *t*‐tests were applied to compare between different groups. Welch's correction was applied if variances between groups were statistically different. ns = not significant. *P < 0.05, **P < 0.01, ***P < 0.001, ****P < 0.0001. Data pooled from two independent experiments.

CoV‐1 and CoV‐2 MVA vaccines induced a similar total magnitude of GC and PB cells in C57BL/6 and in Ig‐humanized mice (Figure 2a). A subset of GC B cells and PBs bound one or more of the RBD tetramers (Figure 2b). CoV‐2 RBD‐binding cells were significantly more frequent in GC but not in PBs following CoV‐2 Spike MVA than in CoV‐1 MVA vaccinated C57BL/6 or Ig‐humanized animals (Figure 2b, c). By contrast, CoV‐1 RBD‐binding cells were more frequent in GC and in PB in C57BL/6 or Ig‐humanized mice given CoV‐1 MVA (Figure 2d).

To enumerate B cells expressing broadly cross‐reacting antibodies, we measured cells that bound three different RBD tetramers from CoV‐1, CoV‐2 Ancestral, and also to CoV‐2 Omicron BA.1 variant, which possesses six additional substitutions of RBD amino acids that were conserved between CoV‐1 and CoV‐2 (G339D, S371L, S375F, N440K, G496S, Y505H). Broadly cross‐reacting GC and PB cells were comparably elicited by CoV‐1 MVA and CoV‐2 MVA immunization (Figure 2e).

### Immunization with CoV‐1 vs CoV‐2 MVA vaccines shows altered humoral immunodominance patterns in a naïve murine and human Ig‐repertoire

**Figure 3:**
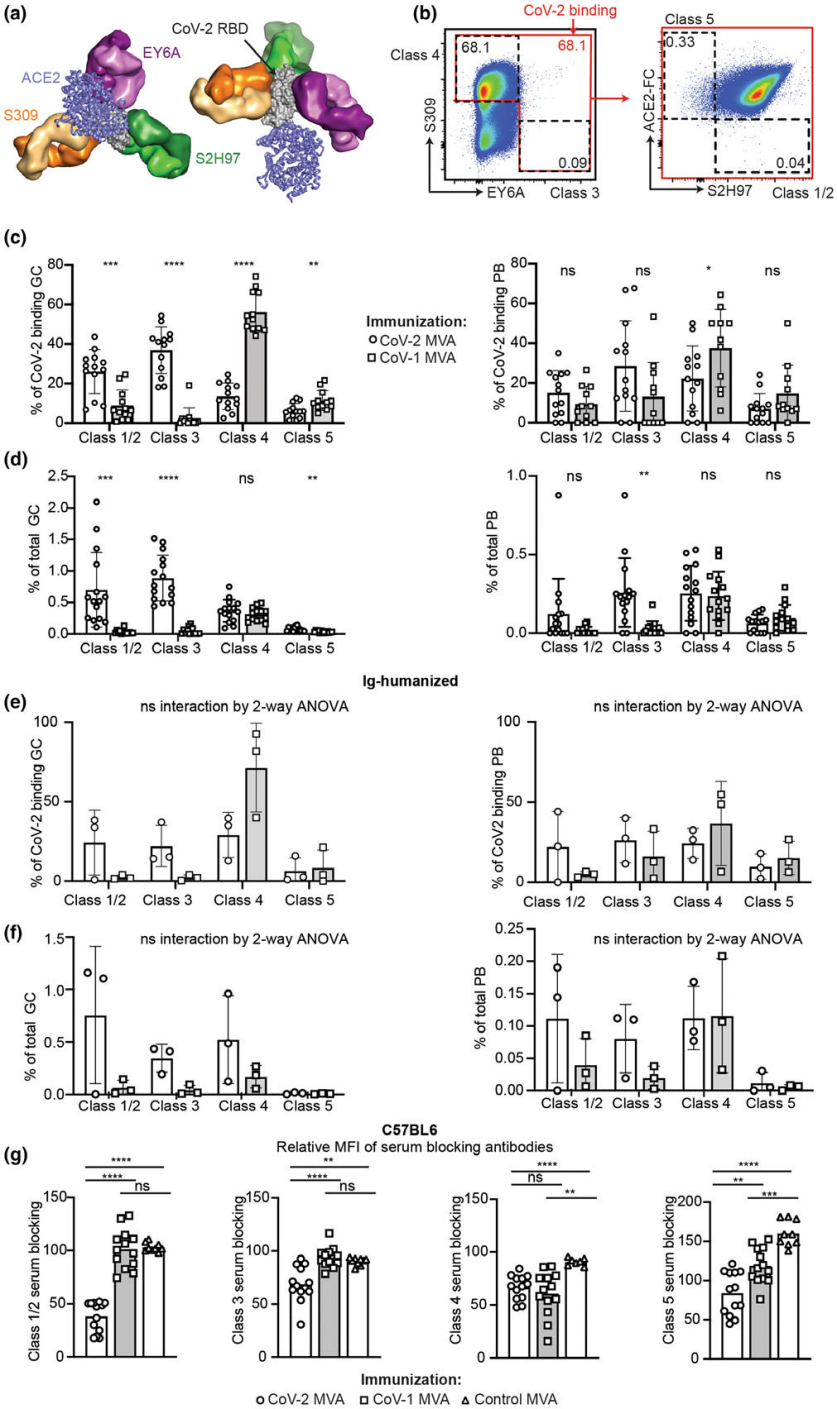
Humoral epitope dominance from immunization of C57BL/6 or Ig‐humanized mice with CoV‐1 or CoV‐2 Spike MVA. C57BL/6 and Ig‐humanized mice were immunized with 2 × 10^8^ PFU IV of CoV‐1, CoV‐2 or control MVA on days 0 and 14 and the immune response examined on day 28. **(a)** Composite model of the CoV‐2 RBD (gray, PDB 7msq) showing the co‐binding of ACE2 to the Class 1/2 epitope (blue, PDB 6m0j), S309 antibody Fab to the Class 3 epitope (orange, PDB 7r6w), EY6A antibody Fab to the Class 4 epitope (purple, PDB 6zcz), and S2H97 antibody Fab to the Class 5 epitope (green, PDB 7m7w). **(b)** Representative flow cytometric plots of spleen B cells from a CR3022 immunoglobulin transgenic mouse expressing membrane IgM encoding the Class 4 antibody on most B cells, showing staining with CoV‐2 RBD followed by a cocktail of each of the fluorescent antibodies and ACE2‐Fc indicated on the Y and Y axis. The gating strategy applied to identify B cells that bind CoV‐2 RBD (red box) through epitopes competed by the indicated fluorescent conjugates (dashed boxes) is shown. **(c)** Percentage of CoV‐2 RBD‐binding B cells from the GC (left) or PB (right) compartment which specifically block staining with fluorescent conjugates against the Class 1/2, Class 3, Class 4, or Class 5 epitope, in individual C57BL/6 mice immunized with CoV‐2 MVA or CoV‐1 MVA. **(d)** Percentage of total B cells from the GC (left) or PB (right) compartment which specifically block staining with fluorescent conjugates against the Class 1/2, Class 3, Class 4, or Class 5 epitope, in individual C57BL/6 mice immunized with CoV‐2 MVA or CoV‐1 MVA. **(e)** Percentage of CoV‐2 RBD‐binding B cells from the GC (left) or PB (right) compartment which specifically block staining with fluorescent conjugates against the Class 1/2, Class 3, Class 4, or Class 5 epitope, in individual Ig‐humanized mice immunized with CoV‐2 MVA or CoV‐1 MVA. **(f)** Percentage of total B cells from the GC (left) or PB (right) compartment which specifically block staining with fluorescent conjugates against the Class 1/2, Class 3, Class 4, or Class 5 epitope, in individual Ig‐humanized mice immunized with CoV‐2 MVA or CoV‐1 MVA. **(g)** Mean Fluorescence Intensity (MFI) of serum antibody response blocking the binding of fluorescent ACE2 (Class1/2) or canonical antibodies to the Class 3, 4, or 5 epitopes to CoV‐2 conjugated to the surface of sheep red blood cells. Data points represent individual mice. Columns show mean ± SEM. A two‐way ANOVA was applied to figures A‐K and a one‐way ANOVA to figure H. If ANOVA revealed significant differences, unpaired *t*‐tests were applied to compare between different groups. Welch's correction was applied if variances between groups were statistically different. ns = not significant *P < 0.05, **P < 0.01, ***P < 0.001, ****P < 0.0001. Data pooled from two independent experiments.

Next, we specifically interrogated the epitope specificity of the RBD response to CoV‐2 MVA or CoV‐1 MVA. Building our established strategies to assess epitope competition,^15^, ^47^ splenocytes were incubated with 500 ng/mL CoV‐2 RBD followed by staining with fluorescent ACE2‐Fc targeting the Class 1/2 (ACE2 binding site) epitope, and fluorescent antibodies against the Class 3 (S309), Class 4 (EY6A), or Class 5 (S2H97) epitopes (Figure 3a). While the Class 1/2 epitope shows only 40% conservation across the CoV‐1 and CoV‐2 RBD, the Class 3, Class 4, and Class 5 epitopes are all highly conserved (>75% conservation) (Supplementary table 1). CoV‐2 RBD‐binding B cells were detected by binding RBD, detected by staining with EY6A or S309. As validated in CR3022 immunoglobulin knock‐in transgenic mice, B cells bearing membrane immunoglobulin that have captured RBD through the Class 4 epitope stain with S309 against the Class 3 epitope but fail to stain with EY6A, since the latter recognizes the occupied Class 4 epitope (Figure 3b).^15^, ^47^ Reciprocally, cells with Ig recognizing the Class 3 epitope would stain with EY6A but fail to stain with S309. Cells recognizing the Class 1/2 epitope would fail to stain with ACE2‐Fc, and cells recognizing the Class 5 epitope would selectively fail to stain with S2H97.

In C57BL/6 (Figure 3c, d, Supplementary figure 2a) and Ig‐humanized (Figure 3e, f, Supplementary figure 2b) mice, CoV‐2 MVA induced a higher proportion of CoV‐2 binding GC B cells and PBs targeting the Class 1/2 and Class 3 epitopes compared to the frequency of cells that blocked the Class 4 or 5 epitopes. The CoV‐2 MVA induced response was more evenly distributed across these epitopes in plasmablasts. In CoV‐1 MVA immunized C57BL/6 or Ig‐humanized mice, a high proportion of GC B cells and plasmablasts that bound CoV‐2 RBD bound via the Class 4 and 5 epitopes. When these epitope specific responses were plotted as a proportion of the total GC and PB response (Figure 3d, f), there was a lower proportion of total PB and GC cells binding the CoV‐2 Class 1/2 or Class 3 epitopes, and equivalent proportions of total PB and GC cells binding the CoV‐2 Class 4 or 5 epitopes.

We next sought to assess the overall capacity of each of these immunizations to induce serum antibodies which bind to these specific epitopes. For these experiments, we developed an assay whereby CoV‐2 RBD was conjugated to SRBC, as previously described,^15^ followed by incubation with fluorescent ACE2‐Fc, S309, EY6A, or S2H97 targeting the canonical Class 1/2, Class 3, Class 4, and Class 5 epitopes, respectively. This allowed us to assess the relative extent to which the immunized mouse serum contained antibodies binding CoV‐2 at each of these specific epitopes and thereby competitively blocking the binding of the fluorescent canonical antibodies for each of these epitopes (Supplementary figure 2c). Consistent with the flow cytometric GC and PB data, CoV‐1 MVA immunized mice produced negligible serum antibodies capable of blocking the binding of ACE2‐Fc or S309 to the CoV‐2 Class 1/2 or 3 epitopes, respectively (Figure 3g). However, CoV‐1 MVA immunization did result in a significant increase in serum antibodies able to block the binding of EY6A or S2H97 to the CoV‐2 Class 4 and Class 5 epitopes, compared to mice given control MVA. In fact, despite the overall lower total serum titers of CoV‐2 antibodies in CoV‐1 immunized mice (Figure 1a), CoV‐1 and CoV‐2 MVA immunization induced similar titers of serum antibodies blocking the Class 4 epitope (Figure 3g).

### Booster immunization with CoV‐1 or CoV‐2 MVA vaccines to induce B cells binding coronavirus RBDs

**Figure 4:**
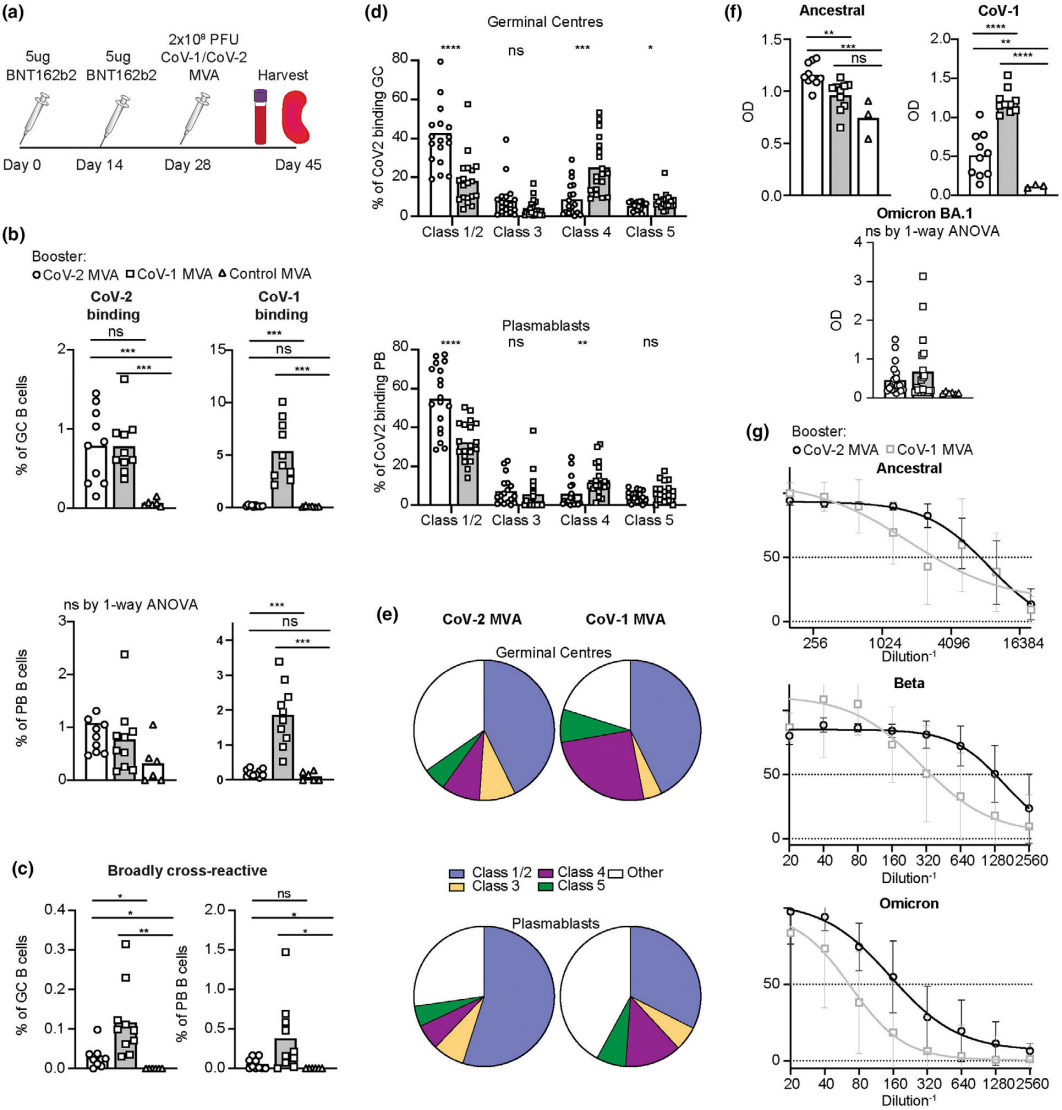
Response to CoV‐1 MVA or CoV‐2 MVA as a booster following primary vaccination with BNT162b2. **(a)** Schematic of experimental plan. C57BL/6 mice were first immunized with 5 μg/mouse IM of CoV‐2 Ancestral Spike mRNA vaccine BNT162b2 on days 0 and day 14. On day 28, mice were boosted with 2 × 10^8^ PFU IV of CoV‐1, CoV‐2 or control MVA, and spleens and blood analyzed on day 45. **(b)** Percentage of GC B cells or plasmablasts (PB) binding to CoV‐2 (left) or CoV‐1 (right) RBD tetramers. **(c)** Percentage of GC (left) or PB (right) cells binding concurrently to CoV‐2 (Ancestral), CoV‐2 (Omicron BA.1), and CoV‐1 RBD tetramers. **(d)** Percentage of CoV‐2 RBD‐binding GC or PB cells in (b) with blocked availability of the Class 1/2, Class 3, Class 4, or Class 5 epitope. **(e)** Pie chart indicating the proportion of the total CoV‐2 RBD‐binding cells blocking the indicated epitopes. **(f)** Optical density (OD) of serum Ig kappa binding to the indicated coronavirus RBDs. Data points represent individual mice. Columns denote arithmetic means. Statistics incorporated one‐way ANOVA. If ANOVA revealed significant differences, unpaired *t*‐tests were applied to compare between different groups. Welch's correction was applied if variances between groups were statistically different. ns = not significant. *P < 0.05, **P < 0.01, ***P < 0.001, ****P < 0.0001. **(g)** Live virus neutralization of the indicated variants causing a cytopathic effect in HEK292T cells following incubation with serum at the indicated dilutions, from C57BL/6 mice vaccinated with CoV‐2 or CoV‐1 MVA. *N* = 10 mice per group for CoV‐1 and CoV‐2 MVA and 6 mice for control MVA. Data pooled from two independent experiments. IC50 was calculated by nonlinear regression (curve fit).

**Figure 5:**
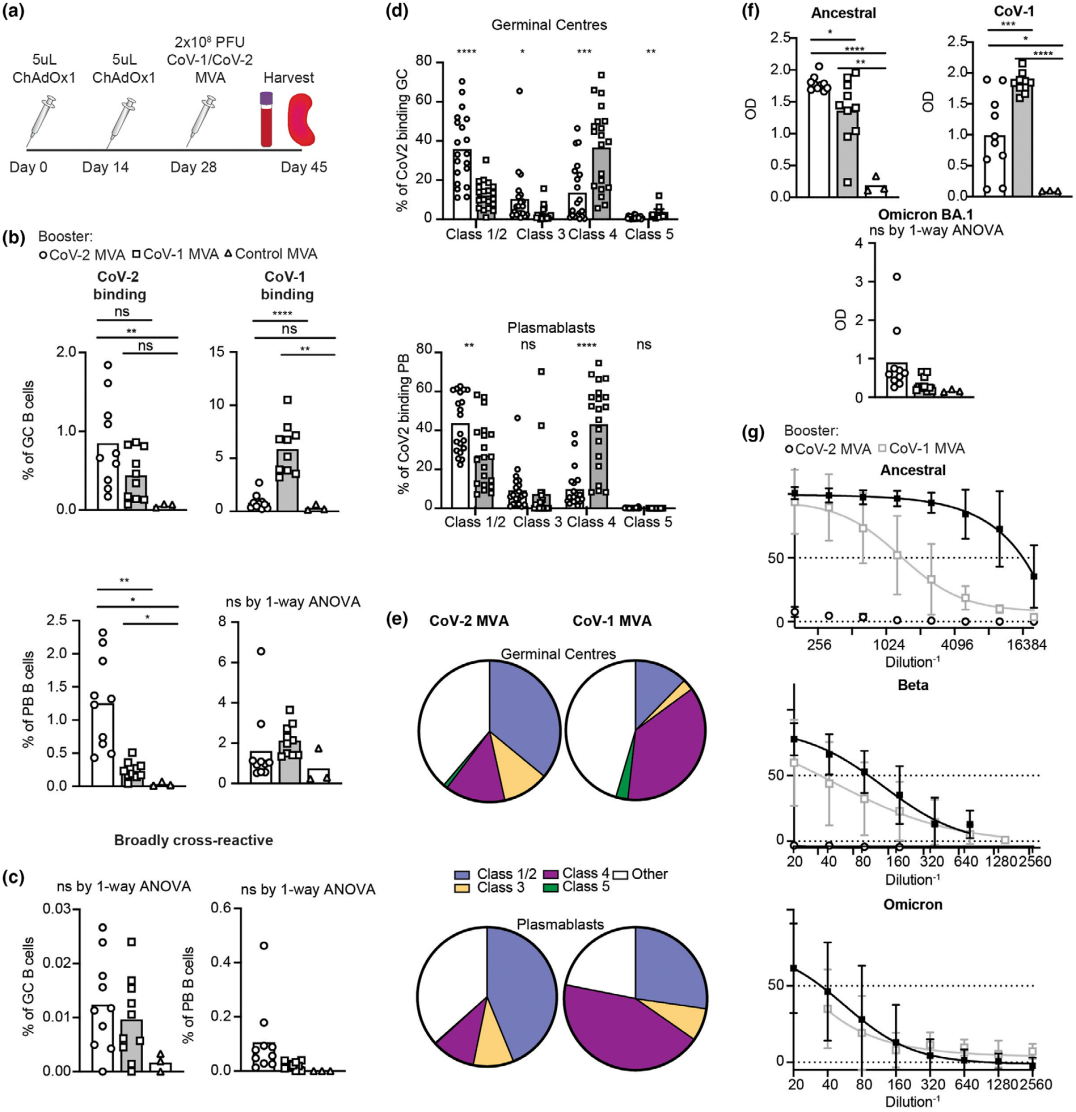
Response to CoV‐1 MVA and CoV‐2 MVA as a booster following primary vaccination with ChAdOx. **(a)** Schematic of experimental plan. C57BL/6 mice were first immunized with 5 μL/mouse IM of a clinically approved CoV‐2 Spike viral vector vaccine (ChAdOx) on days 0 and day 14. On day 28, mice were then boosted with 2 × 10^8^ PFU IV of CoV‐1, CoV‐2 or control MVA and spleens and blood were explored on day 45. **(b)** Percentage of total B cells within the GC (top) or PB (bottom) binding to CoV‐2 (right) or CoV‐1 (left) RBD tetramers. **(c)** Percentage of total B cells within the GC (left) or PB (right) binding concurrently to CoV‐2 (Ancestral), CoV‐2 (Omicron BA.1), and CoV‐1 RBD tetramers. **(d)** Percentage of CoV‐2 RBD‐binding B cells from the GC (top) or PB (middle) compartment which specifically block the Class 1/2, Class 3, Class 4, or Class 5 epitope from C57BL/6 mice boosted with CoV‐1 or CoV‐2 MVA. **(e)** Pie chart indicating the proportion of the total CoV‐2 RBD‐binding response in the GC (top) or PB (bottom) compartment of C57BL/6 mice boosted with CoV‐2 (left) or CoV‐1 (right) MVA. **(f)** Optical density (OD) of serum Ig kappa binding to the indicated coronavirus receptor binding domains. Data points represent individual mice. Columns denote arithmetic means. Statistics incorporated one‐way ANOVA. If ANOVA revealed significant differences, unpaired *t*‐tests were applied to compare between different groups. Welch's correction was applied if variances between groups were statistically different. ns = not significant. *P < 0.05, **P < 0.01, ***P < 0.001, ****P < 0.0001. **(g)** Live viral neutralization of the indicated variants of HEK292T cells following incubation with serum at the indicated dilutions, from C57BL/6 mice vaccinated with CoV‐2 or CoV‐1 MVA. *N* = 10 mice per group for CoV‐1 and CoV‐2 MVA and 6 mice for control MVA. Data pooled from two independent experiments.

Next, we sought to expand these findings into a system to better recapitulate the current global vaccine landscape. For these experiments, mice were pre‐immunized with either two doses 2 weeks apart of 5 μg BNT162b2 or 5 μL ChAdOx1 clinically approved mRNA and adenoviral vector CoV‐2 Ancestral Spike vaccines, respectively, that have been utilized in the majority of the world's population.^48^, ^49^, ^50^ Two weeks later, these primed mice were boosted with 2 × 10^8^ PFU of CoV‐1 or CoV‐2 MVA or control MVA (Figures 4a and 5a). For these experiments, the timing of boosting was selected to explore the effect of a secondary booster at peak primary GC response^51^, ^52^, ^53^ similar to approaches validated for germline targeting and guiding vaccines.^54^ While the CoV‐1 MVA booster led to a significantly higher proportion of CoV‐1 RBD‐binding B cells in the GC and PB compartment, the equivalent effect on CoV‐2 binding B cells was far less marked and largely not significant (Figures 4b and 5b). The CoV‐1 booster led to an equivalent or greater proportion of GC or PB B cells cross‐reacting between all CoV‐1, CoV‐2 (Ancestral), and CoV‐2 (Omicron BA.1) (Figures 4c and 5c). When examining the epitope distribution profile of the CoV‐2 binding B cells within these compartments, similar to what was seen in the MVA primary immunization experiments, CoV‐2 MVA booster induced more B cells binding the Class 1/2 and 3 epitopes, and a lower proportion of B cells binding the Class 4 and Class 5 epitopes. However, it should be noted that the effect of CoV‐1 MVA vaccines induced a lower proportion of the GC and PB response against the Class 3 epitope was much less pronounced when these vaccines were utilized as a booster following BNT162b2 or ChAdOx1 (Figures 4d, e and 5d, e).

In the serum, mice boosted with CoV‐2 MVA had significantly increased serum antibody titers to the CoV‐2 Ancestral strain with the equivalent effect holding true for CoV‐1 MVA boost and CoV‐1 serum antibody titers (Figures 4f and 5f). Interestingly, despite the homology of the Ancestral CoV‐2 to Omicron BA.1, compared to CoV‐1, mice boosted with CoV‐2 MVA had no significant increase in the total serum binding Omicron BA.1 (Figures 4f and 5f). Similarly, when the protective capacity of the serum was assessed by live virus neutralization in HEK292T cells, overall CoV‐2 MVA did not induce significantly increased neutralizing protective antibodies against the Beta or Omicron BA.1 variants, compared to CoV‐1 MVA (Figures 4g and 5g).

### CoV‐1 booster recruits in *de novo* B cells into the response

**Figure 6:**
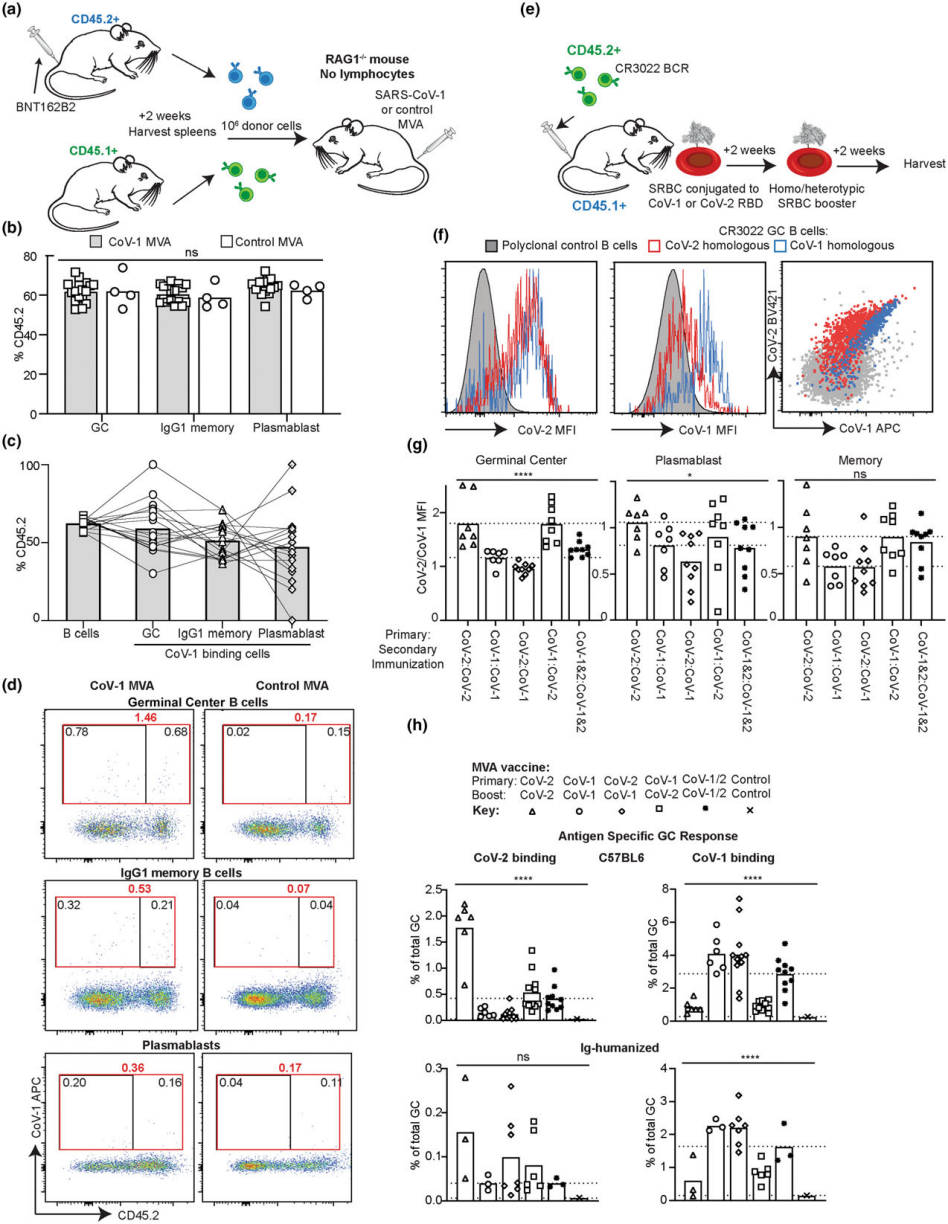
Effect of immune imprinting on response to CoV‐1 or CoV‐2 MVA. **(a)** Schematic of experimental plan for B‐D. C57BL/6 (CD45.2) mice were immunized with 5 μg/mouse IM of CoV‐2 Spike mRNA vaccine BNT162b2 on day −14 and spleen cells harvested on day 0. These were mixed with an equal number of spleen cells from naïve CD45.1 congenic mice and 2 × 10^6^ cells transferred to RAG1^−/−^ mice, and the recipients immunized with 2 × 10^8^ PFU CoV‐1 or control MVA. Splenic response was analyzed on day 14 after transfer. **(b)** Proportion of GC, IgG1 memory or PB compartment derived from the CD45.2 donor mice in mice immunized with CoV‐1 (gray columns, squares) or Control MVA (white column, circles). GC and PB gating were performed as per Figure 1 with the addition of CD45.2 and CD45.1 antibody staining. IgG1^+^ memory B‐cell response was also assessed as CD45.2^+^ B220^+^ CD138^−^ CD38^+^ CD95‐ IgG1^+^ cells. **(c)** Proportion of CD45.2 cells among all B cells (left column) or of CoV‐1 RBD‐binding GC or memory B cells or plasmablasts. Lines connect data points derived from the same mouse. Data points represent individual mice. *P < 0.05, **P < 0.01, ***P < 0.001, ****P < 0.0001. Data pooled from two‐three independent experiments. Columns represent arithmetic mean. **(d)** Representative flow cytometric plots of the splenic response at day 14 showing the proportion of CoV‐1 binding B cells that were derived from the CD45.2 congenic BNT162b2 immunized or CD45.1 congenic naïve donor mice. **(e)** Schematic of experimental plan for B‐C. Splenocytes from CD45.2 congenic C57BL/6 mice genetically engineered to express the human CoV‐1 and CoV‐2 cross‐reactive BCR, CR3022, were adoptively transferred into CD45.1 congenic recipient mice. Mice were then immunized with 200 000 SRBC conjugated to CoV‐1 or CoV‐2 RBD on day 0 followed by heterotopic or homotopic immunization on day 14 and with spleens and blood analyzed on day 28. **(f)** Representative flow cytometric plots from the CoV‐2 (red), or CoV‐1 (blue) immunized mice described above showing the CR3022 B GC cells binding of CoV‐1 and CoV‐2 RBD tetramers. Polyclonal B cells from a C57BL/6 mouse immunized with unconjugated SRBC are also shown for reference. **(g)** Relative ratio of CoV‐2/ CoV‐1 mean fluorescent intensity (MFI) of the CR3022 GC B cells, with a higher ratio indicating higher CoV‐2 binding relative to CoV‐1. GC and PB cells were determined as per Figure 1 gating only on the CD45.2^+^ population. Memory B cells were determined as the CD45.2^+^ non‐GC or PB cells. **(h)** C57BL/6 or Ig‐humanized mice were immunized with 2 × 10^8^ PFU of CoV‐1 or CoV‐2 or an equal mixture of CoV‐1 and CoV‐2 MVA on day 0. On day 14, mice were given a homologous or heterologous MVA immunization or the mixture again. Splenic response was analyzed on day 28 to measure the % of GC B cells binding CoV‐2 (left) or CoV‐1 (right) RBD tetramers.

Adoptive transfer experiments were used to better understand what fraction of the CoV‐1 recall response consisted of cells that had experienced the Ancestral CoV‐2 as opposed to recruitment of naïve B cells. Again, the timing of boosting was selected to focus exploration on the response of B cells currently responding to the primary antigen.^51^, ^52^, ^53^ C57BL/6 mice were immunized with 5 μg/mouse BNT162b2, and 2 weeks later, spleens from these mice were mixed with an equal number of splenocytes from a naïve C57BL/6 CD45.1 congenic mouse not pre‐immunized with BNT162b2. The equal mixture of primed and naïve splenocytes, distinguished by CD45 allelic markers, was transferred into RAG1^−/−^ mice, lacking lymphocytes, and the recipients immunized with 2 × 10^8^ PFU CoV‐1 MVA or control MVA (Figure 6a). In the CoV‐1 MVA immunized recipients, CoV‐1 RBD‐binding GC or IgG1 memory B cells or plasmablasts were drawn from the primed CD45.2 donor no more frequently than CD45.2^+^ B cells in the recipient animals (Figure 6b–d). When explored using a dual tetramer staining strategy, the CoV‐1 binding B cells within these compartments showed minimal evidence of cross‐reactivity (Supplementary figure 3a). Thus, MVA expressing the CoV‐1 spike effectively recruits new B cells into making CoV‐1 RBD‐binding antibodies even in the presence of CoV‐2 primed cells.

### CoV‐1 and CoV‐2 cross‐reactive B cells undergo affinity maturation toward the booster antigen

Given the above findings, that the secondary response to CoV‐1 vaccines did not predominantly consist of B cells with prior antigenic experience to CoV‐2, we next sought to explore the fate of cross‐reactive B cells, recognized to constitute a small but re‐occurring component of the vaccination response to both CoV‐1 or CoV‐2 immunizations.^15^ For these experiments, 100 000 CD45.2^+^ B cells expressing the CR3022 BCR, with cross‐reactive affinity to CoV‐1 and CoV‐2 (1.3 and 25 nM, respectively), were transferred into CD45.1^+^ recipients.^47^, ^55^, ^56^ Following the transfer of CR3022 specific B cells, recipient mice were immunized with SRBC, serving as a strong T‐cell adjuvant,^57^ conjugated to either CoV‐1 or CoV‐2 RBD or a combination of the two. Two weeks later, recipient mice were immunized with a secondary SRBC immunization consisting of a homologous or heterologous antigenic combination (Figure 6e).

As CR3022 B cell efficiently bind to both CoV‐2 and CoV‐1 tetramers, to detect subtle differences in B‐cell affinity, internally corrected for staining affinity per mouse, we assessed the relative ratio of CoV‐2 vs CoV‐1 Mean Fluorescence Intensity (MFI) (Figure 6f). This revealed a relative MFI shift toward the most recently immunized antigen, with the CR3022 B cells from mice given CoV‐2 and CoV‐1 conjugated SRBC concurrently having a more intermediate CoV‐2/CoV‐1 MFI ratio (Figure 6g).

### Exploration of the reciprocal capacity of CoV‐1 and CoV‐2 primary and secondary responses

The above findings indicated that mice immunized with CoV‐2 primary response could respond to a CoV‐1 booster, predominantly through recruitment of *de novo* B cells and through further affinity maturation of B cells already cross‐reactive to both antigens. We next aimed to understand whether CoV‐1 immunized animals boosted with CoV‐2 were equally able to respond to CoV‐2 as the reciprocal immunization strategy. For these experiments, C57BL/6 or Ig‐humanized mice were vaccinated first with either 2 × 10^8^ PFU CoV‐2 or CoV‐1 MVA followed two weeks later by a homologous immunization with CoV‐2 MVA or a heterologous immunization with CoV‐1 MVA (Figure 6h).

Consistent with the findings above, boosting with CoV‐1 was equally capable inducing CoV‐1 binding B cells in the GC, regardless of whether the primary immunization was CoV‐1 or CoV‐2. Mice concurrently immunized with CoV‐1 and CoV‐2 MVA at 1 × 10^8^ PFU at both timepoints had an intermediate phenotype, consistent with the lower dose of immunizing antigen. However, boosting mice with CoV‐2 MVA was less capable of inducing a CoV‐2 binding GC B cell response if the primary immunization was with CoV‐1 MVA, compared to a primary CoV‐2 homologous immunization.

As expected for this timepoint, only two weeks postbooster, these changes were not yet fully reflected in the serum response, which more closely reflected the primary immunization (Supplementary figure 3b). Of note, the CoV‐1 and CoV‐2 MVA vaccines when immunized as a primary and homologous booster immunization induced a similar magnitude of serum antibody titers to each other, suggesting that the above differences in the heterologous GC response between CoV‐1 and CoV‐2 were not likely caused by any differences in the immunogenicity of the CoV‐1 and CoV‐2 MVA particles.

These findings show that while both the CoV‐1 and CoV‐2 MVA vaccines were equally potent at inducing serum antibodies, the capacity of GC B cells to respond to a secondary heterologous immunization with CoV‐1 MVA was not equally matched by the capacity of GC B cells to respond to a secondary heterologous immunization with CoV‐2 MVA.

## DISCUSSION

In this study, we have shown using a well‐studied and physiologically relevant human vaccination system that CoV‐1 and CoV‐2 Spike induce distinct B cell epitope dominance patterns in the GC which extend into plasmablast and serum response. This discovery builds on and expands from our previous work showing that CoV‐1 RBD conjugated to foreign erythrocytes induced a higher proportion of the CoV‐2 RBD response to target the Class 4 epitope.^15^ The Class 4 epitope is of importance in vaccine development as it shows a high degree of sequence conservation across the coronavirus lineage.^15^, ^58^, ^59^ However, Omicron variants diminish the neutralizing capacity of Class 4 antibodies^22^ highlighting that this epitope alone cannot be used to evaluate a vaccine capacity to induce conserved B cell response. This study has therefore extended our understanding beyond just the Class 4 epitope to concurrently evaluate the response to the ACE2 binding motif that incorporates the Class 1 and Class 2 epitopes,^13^ as well as other highly conserved epitopes including the Class 3 epitope, as represented by the canonical S309 antibody^19^ and the more recently described conserved Class 5 epitope.^14^ This together allowed us to paint a complex picture of the differencing epitope dominance patterns induced by CoV‐1 and CoV‐2 MVA and highlight implications for vaccine development. In addition to directing an increased proportion of the response against the Class 4 epitope, CoV‐1 MVA was more capable of directing the B‐cell response to target the Class 5 epitope. Class 5 antibodies target highly conserved sites and show considerable evidence of cross‐protective immunity that is maintained against the Omicron subvariants.^11^, ^14^, ^60^, ^61^ While CoV‐1 immunization skewed the response toward the Class 4 and 5 responses, CoV‐2 MVA immunizations resulted in a B‐cell response predominantly targeting the Class 3 and ACE2 receptor binding motif that incorporates the Class 1 and Class 2 epitopes.^13^, ^18^ These findings are consistent with those seen in human serum responses^10^, ^62^, ^63^ but have not been previously explored to a physiological vaccine in mouse and humanized repertories at a single cell resolution within the GC compartment. Because the ACE2 binding motif is extraordinarily variable, Class 1/2 antibodies are frequently high‐potency but low‐breadth. However, there are several examples of antibodies targeting the Class 2 epitope that still retain some potency against immune evasive strains, although usually several orders of magnitude lower than their potency against Ancestral CoV‐2.^64^, ^65^ Antibodies from the Class 3 group show cross‐reactivity between SARS‐CoV‐1 (CoV‐1) and CoV‐2 and can cross‐neutralize Omicron BA.1, and however, they have greatly diminished capacity to neutralize later omicron variants.^66^, ^67^, ^68^ Together, these findings produce a more complex understanding of the distinct epitope dominance patterns induced by CoV‐1 and CoV‐2 vaccines that cannot be simplistically distilled to one or the other having a distinct advantage in vaccine design but instead highlight the complex interplay that must be interpreted in intelligent antigen selection for vaccine design.

To extend the physiological relevance of these findings, we also explored the capacity of CoV‐1 vaccines as a booster immunization following previous immunization with the clinically approved BNT162b2 mRNA or ChAdOX adenoviral vector vaccines. Overall, the immunodominance profile of CoV‐1 MVA vaccines as a booster vaccine was like that of a primary immunization. A CoV‐1 MVA, as opposed to a CoV‐2 MVA booster, resulted in a higher proportion of the GC and PB response targeting the Class 4 and Class 5 epitopes, and a lower proportion of the CoV‐2 response targeting the Class 1/2 and Class 3 epitopes. Some of the comparative effects seen when CoV‐1 MVA was used as a primary vaccination, such as the decreased total CoV‐2 binding antibody titer or neutralization potency and the decreased prevalence of the Class 3 epitope, were less marked when CoV‐1 was utilized as a booster vaccine. This was particularly evident when the CoV‐1 MVA vaccine was used as a booster following the BNT162b2 vaccine. This may relate to the BNT162b2 vaccine having already induced a ceiling titer of Class 3 antibodies, therefore highlighting the benefits of diversifying the humoral response against the Class 4 and Class 5 epitopes. Accordingly, CoV‐1 MVA booster following BNT162b2 induced a greater number of broadly cross‐reactive B cells compared to a CoV‐2 booster. This is consistent with data produced by Hu *et al*., who also showed that a CoV‐1 viral vector booster vaccine was superior to an equivalent CoV‐2 vaccine at inducing a cross‐neutralizing response following two prior vaccinations with BNT162b2^7^ and is especially of impact considering that third boosters with BNT162b2 are already known to increase the breadth of the response.^69^ Together, this data provided evidence that the CoV‐1 MVA induced a distinct epitope dominance pattern as a primary immunization pattern and that this epitope dominance pattern was still evident in the GC and PB compartment when it was used as a booster immunization following clinically approved CoV‐2 Spike vaccines.

To better dissect why the epitope dominance patterns of CoV‐1 as a primary immunization were equally evident when it was used as a booster, and to explore the role of memory B cells in the booster response, we developed an adoptive transfer system, transferring CoV‐2 experienced and naïve B cells into donor mice and immunizing with CoV‐1 MVA. Interestingly, these adoptive transfer studies indicated that CoV‐2 experienced B cells did not show any selective advantage in the CoV‐1 specific booster GC, IgG1 memory, or plasmablast response. This differs from the findings seen with Omicron booster vaccines in both humans^41^, ^42^, ^43^, ^44^, ^45^ and mice^30^ where the response predominantly consists of recalled cross‐reactive B cells. Given the lack of sequence homology between CoV‐1 and CoV‐2 compared to CoV‐2 Ancestral and Omicron (97% vs 75% homology), this finding is consistent with the hypothesis that antigenic distance between the secondary antigen would result in predominantly *de novo* B cells in the secondary response.^7^, ^37^, ^38^, ^39^, ^40^ However, it cannot be excluded that an increased proportion of cross‐reactive B cells would have been recruited if the immunization schedule had been extended to facilitate improved recruitment of the primary response into the memory compartment.

The hypothesis that CoV‐1 *de novo* B cells can be efficiently recruited following CoV‐2 primary vaccination was further supported by experimental evaluation of the antigen‐specific GC response to CoV‐1 and CoV‐2 following homologous or heterologous boosts with CoV‐1 or CoV‐2 MVA. These experiments revealed that C57BL/6 or Ig‐humanized mice immunized with CoV‐2 followed by CoV‐1 MVA could mount a similar CoV‐1 GC response as mice given two homologous immunizations with CoV‐1 MVA. Interestingly, the converse was not equally true. Mice immunized with a CoV‐1 primary immunization followed by a CoV‐2 boost did not induce as many CoV‐2 binding B GC cells as mice given homologous primary and booster immunizations with CoV‐2 MVA. Together, these findings suggest that while CoV‐1 and CoV‐2 MVA were both effective as primary vaccines, the capacity of CoV‐1 MVA utilized as a booster vaccine following CoV‐2 primary immunization was not reciprocally matched by the capacity of CoV‐2 MVA as a heterologous booster vaccine. This could be a consequence of the improved capacity of the CoV‐1 vaccines to induce a higher magnitude of response against conserved sites, paradoxically also enhancing negative antibody feedback that diminishes the secondary response to CoV‐2.^70^, ^71^ The fact that the secondary homologous immunizations with CoV‐1 were not equally diminished reduces the likelihood of this hypothesis. Regardless of the mechanism, these findings could have implications for a future spillover of CoV‐1‐like sarbecoviruses and indicate the future booster immunizations with a CoV‐1 vaccine will be capable of inducing a strong *de novo* response that overcomes immune imprinting. These findings challenge previous hypotheses by ourselves and others that CoV‐1 vaccines could have served as an improved primary vaccine antigen.^15^, ^72^ They are especially intriguing considering that SARS‐CoV‐1 survivors immunized with CoV‐2 vaccines showed increased breadth of response.^6^ However, this could be driven by factors present in a SARS‐CoV‐1 infection setting that are not recapitulated with vaccination. This finding that immune imprinting does not have equal directionality in both directions is intriguing and implies that immune imprinting may be driven by other factors beyond just antigenic distance that have not yet been fully elucidated.^7^, ^39^, ^40^ It is unclear whether this results in a stronger immune bias imprinted from the primary immunization or a reduced capacity of the secondary immunization to overcome this bias.

Although cross‐reactive B cells to both CoV‐1 and CoV‐2 may only constitute a small minority of the response, these B cells may have a distinct importance in inducing cross‐protective immunity. Many studies have evaluated CoV‐1 and CoV‐2 cross‐reactive antibodies as a small but distinct proportion of the immune response to either antigen.^14^, ^15^, ^19^, ^55^, ^64^, ^73^, ^74^, ^75^, ^76^ While these cells may constitute an important aspect of the acute response to secondary antigens with a divergent antigenic distance to the primary immunization, before the *de novo* response has sufficient time to expand and affinity mature, it remains to be explored whether these cross‐reactive antibodies would be capable of longer term recruitment into the GC, memory, or plasmablast compartment and if they would be capable of maturing toward the secondary antigen. By exploring the relative CoV‐1 and CoV‐2 binding of CR3022 transgenic B cells, with a defined cross‐reactive affinity to CoV‐1 and CoV‐2, we were able to show that cross‐reactive B cells increase binding in the GC toward the most recently exposed antigen. This indicates that cross‐reactive B cells could mature further against multiple heterologous antigenic scenarios, even against targets with a divergent antigenic distance like CoV‐1 and CoV‐2. Interestingly, the CoV‐2:CoV‐1 MFI from CR3022 B cells immunized with CoV‐2 followed by CoV‐1 was lower than that immunized with homologous CoV‐1 primary and booster. This is suggestive that despite their high pre‐existing affinity to CoV‐1, the heterologous immunization strategy may have opened additional pathways for affinity maturation that were not evident with homologous boosts. This finding is reminiscent of what has been seen in transgenic B cells, which can acquire increased affinity toward a foreign antigen, when they are forced to concurrently navigate self‐reactivity,^77^, ^78^ and this approach has been incorporated in vaccine strategies against conserved sites in influenza^79^ and HIV vaccination approaches.^80^, ^81^

Although this data indicates that such B cells could play a role in repeated heterologous immunizations, the predefined affinity and frequency of these B cells inherently present in a B‐cell receptor transgenic system, restricts the applicability of these findings in different vaccine settings. Due to the species divergence of CoV‐1 and CoV‐2 RBD, which only share 75% homology, antibodies which cross‐react to both RBDs have been theorized as more likely to target conserved sites shared between the two viruses.^15^, ^82^, ^83^ As such, the proportion of antibodies cross‐reacting between both antigens has been proposed as a proxy for the proportion of the vaccine response targeting conserved sites. Given the number of unique mutations specific to the Omicron variant and that many cross‐reactive antibodies against CoV‐1 and CoV‐2 lose their potency against Omicron,^22^ the addition of Omicron to these assays allow for the selection of antibodies with a higher conservation potential against future variants.^83^ When taken in concert with the above findings in the polyclonal system that a CoV‐1 MVA booster following BNT162b2 induced a greater number of broadly cross‐reactive B cells compared to a CoV‐2 booster, these findings together suggest that a CoV‐1 booster can cause further expansion in numbers, as well as affinity, of cross‐reactive cells with existing high affinity toward the vaccine antigen.

Together this body of work adds important insights for the capacity of next generation antigens, including CoV‐1, to serve as next generation booster vaccine strategies against sarbecovirus targets.

## METHODS

### Mice

All mice used were housed at Australian BioResources and held at the Garvan Institute of Medical Research in specific pathogen‐free environments. TRIANNI mice (San Francisco, CA, USA), obtained with permission from Hans‐Martin Jäck, expressing a fully humanized variable antibody repertoire.^15^, ^46^ Transgenic mice expressing the rearranged heavy and light chain genes of CR3022 were produced by the Mouse Engineering Garvan/ABR (MEGA) Facility using CRISPR/Cas9‐mediated gene targeting in C57BL/6J embryos.^15^, ^47^, ^56^ Rearranged CR3022 L‐VDJ heavy and L‐VJ kappa exons were linked to 5' mouse *Ighv* and *Igkv* promoters and inserted 3' of the endogenous *Ighj4* and *Igkj5* segments, respectively. Mice hemizygous for both targeted loci were utilized for analysis. Mice were used between the ages of 7–12 weeks, with both male and female mice included. Mice were excluded if they showed any evidence of disease prior to recruitment. The Garvan Animal Ethics or Sydney Local Health District Animal Welfare Committees approved all mice protocols and procedures.

### Recombinant protein reagents

CoV‐2 variant and CoV‐1 RBD proteins and ACE2‐Fc were ordered from Sinobiological. To make fluorescent RBD tetramers, biotinylated versions of the relevant RBD proteins (Sinobiological) were conjugated to the relevant streptavidin fluorophores at a 4:1 molar ratio for 1 h at 4 degrees. Excess biotinylated protein that was not coupled to streptavidin was removed by size exclusion through 30KMWCO Amicon Ultracel centrifugal columns (Merck Millipore Ltd). All recombinant protein fluorophores were titrated prior to use using B cells from CR3022 knock‐in mice.

Antibodies were transiently expressed as human IgG1 in HEK293 cells using standard plate transfection and the Expi system (Life Technologies) and purified with protein G Sepharose (GenScript) according to the manufacturers' recommendations. Buffer exchange was performed using GenScript desalting preparation columns. Quality control of proteins included SDS‐PAGE and Western blots. Samples that failed to meet QC or were unable to be expressed at high concentrations (<0.01 mg/mL) were removed from the analysis. Prior to use, 100 μg of EY6A, S309, S2H97 was conjugated using commercial Invitrogen antibody conjugation kits.

### Modified vaccinia virus

CoV‐2 Ancestral and CoV‐1 Spike MVA viruses used in this study have been described previously.^31^ Both the CoV‐1 and CoV‐2 Spike MVA constructs utilized Spike protein modified by stabilization of the prefusion structure by proline substitutions (K986P, V987P) to the S2 domain of the CoV‐2 Spike. The MVA virus was produced by homologous recombination, clonally purified by repeated plaquing, and physically purified by sedimentation through sucrose. Experiments involved injection of 2 × 10^8^ PFU IV by retroorbital injection.

### Sheep red blood cells (SRBCs) conjugation and binding assays

Conjugation was performed as described previously.^77^ Sheep Red Blood Cells (SRBCs) were washed in phosphate‐buffered saline (PBS) three times by centrifugation at 2300 rpm (1111 g) for 5 min at 4°C and then once in conjugation buffer. SRBCs were then resuspended in a final volume of 1 mL conjugation buffer containing 10–30 μg/mL of protein for conjugation. The solution was mixed on a platform rocker on ice for 10 min. About 10 mg N‐(3‐Dimethylaminopropyl)‐N‐ethylcarbodiimide hydrochloride (EDCI) (Sigma) was then added, and the solution was mixed for a further 30 min on ice. SRBCs were then washed four times in PBS. Confirmation of successful conjugation was performed by flow‐cytometric analysis of SRBCs using fluorescently conjugated S309. Mice were immunized with 200 000 SRBCs per mouse intravenously via retroorbital injection.

For assays to assess the binding of expressed antibodies to CoV protein conjugated SRBCs, 10 000 SRBCs were aliquoted into individual wells of a 96 well plate. Antibodies and ACE2 were titrated against CoV‐2 RBD‐conjugated SRBCs to determine a sub‐saturating dilution of the fluorescent antibody to be used to measure competition with other unconjugated antibodies. The mean fluorescent intensity of binding of these fluorescent antibodies to CoV‐2 RBD‐conjugated SRBCs was measured after incubating the SRBCs with mouse serum at a 1:50 dilution for 30 min on ice, compared to the MFI with no competing antibody measured in parallel. % inhibition = MFI test/MFI no competitor × 100.

### Flow cytometry

On the day of harvest, organs were collected into PBS with 1% Bovine Serum Albumin (BSA) (Bovogen), cell suspensions passed through a 70 μm cell strainer (Falcon, Corning, NY, USA) and centrifuged 1500 rpm (440 g) for 5 min at 4°C. Fc receptors were blocked with unlabeled anti‐CD16/32 (eBioscience or BD) before staining.

Single cell suspensions were labeled with the following anti‐mouse antibodies: IgG1‐BUV395 (10.9, BD Biosciences), Fas‐PeCy7 (Jo2, BD Biosciences), CD38‐BV510 (90/CD38, BD Biosciences) CD4 ‐AF700 (RM4‐5, BD Biosciences), B220‐BUV737 (RA3‐6B2, BD Biosciences), CD11b‐PE (M1/70, BD Biosciences), TCRB‐BV711 (H57‐597, BD Bioscience), and IgD APCCy7 (11‐26c.2a, BioLegend). Live dead discrimination was performed with 7AAD (BioLegend). B cells were determined as TCRB^−^, CD4^−^, CD11b^−^, and B220^+^ cells. Germinal centers were identified as Fas^+^, CD38^−^, IgD^−^.

Cells were filtered using 35 μm filter round‐bottom FACS tubes (BD Pharmingen) immediately before data acquisition on a LSR II analyzer (BD Pharmingen). Forward‐ and side‐scatter threshold gates were applied to remove red blood cells and debris and approximately 2–5 × 10^6^ events were collected per sample. Cytometer files were analyzed with FlowJo software (FlowJo LLC, Ashland, Oregon, USA).

For flow cytometric epitope binding assays, spleens from immunized mice were incubated with CoV‐2 RBD at 200 ng/mL in 1% BSA. While incubating with the other surface stains above samples were incubated with fluorescently conjugated S309 (Class 3), EY6A (Class 4) and S2H97 (Class 5) as well as ACE2‐Biotin (class 1/2) (Sinobiological). Between stains samples were washed by the addition of 100 μL PBS and centrifuged at 2300 rpm (1111 g) for 1 min at 4°C.

### Serum ELISA

High‐binding plates (Corning, Corning, NY, USA) were coated with the relevant CoV recombinant proteins at 5 μg/mL in a sodium bicarbonate buffer (0.3% NaHCO_3_, 0.2% NaCO_3_, 0.01% MgCl_2_) overnight at 4 degrees. Wells were then blocked with 1% BSA in PBS for 1 h at 35°C, followed by incubation with mouse serum. Bound serum antibody was quantified using a monoclonal antibody to mouse IgK‐biotin (187.1, BD Biosciences) followed by streptavidin‐alkaline phosphatase (Sigma) and SigmaFast P‐ Nitrophenyl Phosphate tablets (Sigma).

### High‐content live CoV‐2 neutralization assay

The NAb assay was performed as previously described.^22^ Clonal HAT‐24 cells were generated by transducing lentiviral particles into HEK292T (Thermo Fisher, R70007) cells to stably express ACE2 and TMPRSS2 receptors. These were then trypsinized and resuspended in DMEM‐5% fetal bovine serum (FBS) and stained with Hoechst‐33 342 (5% v/v) live nuclear dye (Invitrogen, R37605) and seeded in a 384‐well plate at 1.6 × 104 cells per well. Test sera were serially diluted (twofold) with DMEM‐5% FBS, and an equal volume of SARS‐CoV‐2 virus solution at twice the median lethal dose was added to the diluted sera. Sera‐virus mixture was incubated for 1 h at 37°C before 40 μL per well was added to the plated cells. Plates were incubated for 20 h at 37°C, 5% CO2 before enumerating nuclear counts with high‐content fluorescence microscopy using Cytiva InCell Analyzer HS2500 and IN Carta software. Percentage of virus neutralization was calculated with the formula %*N* = (D‐(1‐Q)) × 100/D (where Q = nuclei count/average count for uninfected controls, and D = 1‐Q = average count for infected controls). Sigmoidal dose–response curves and interpolated IC50 values (reciprocal dilution at which 50% neutralization is achieved) were determined using Sigmoidal, 4PL model of regression analysis in GraphPad Prism (GraphPad Software, USA).

### Quantification and statistical analysis

GraphPad Prism 8 (GraphPad Software, San Diego, USA) was used for data analysis. A one‐ or two‐way ANOVA was applied to all analyses involving comparisons with more than two groups. If the ANOVA showed significance, then pairwise *t*‐tests were used to compare between two groups. When the data were normally distributed, an unpaired Student's *t*‐test was performed for analysis. When data were not normally distributed Welch's correction was applied. For all tests, *P* < 0.05 was considered as being statistically significant. Unless otherwise stated, error bars represent standard error of mean. For all figures, data points indicate individual mice. * represents *P* < 0.05, ** represents *P* < 0.01, *** represents *P* < 0.001, and **** represents *P* < 0.0001.

## CONFLICT OF INTEREST

The authors have no conflict of interest to declare.

## Supporting information


Supplementary Figure 1.
**Supplementary figure 2**.
**Supplemnetary figure 3**.

Supplementary table 1.

## Data Availability

The data that support the findings of this study are available in the supplementary material of this article.
