# Supplementary material for: Humoral epitope dominance and immune imprinting by SARS‐CoV‐1 and SARS‐CoV‐2 vaccines
Source: Immunol Cell Biol. 2026 Jan 6;104(2):150–67. doi: 10.1111/imcb.70072 (PMC12872407; doi:10.1111/imcb.70072)
Supplement: Supplementary file 1 — Supplementary Figure 1. Supplementary figure 2. Supplemnetary figure 3. [file IMCB-104-150-s001.pdf]

Supplementary material:

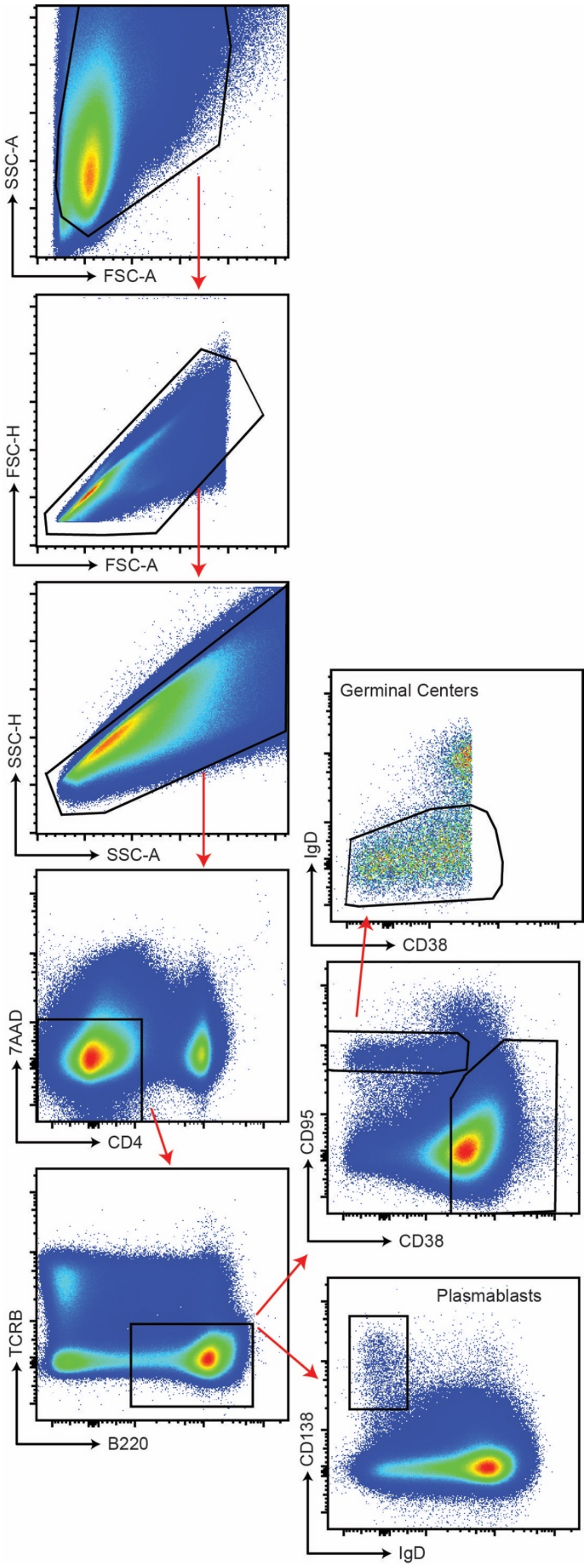

Figure S1. Representative flow cytometric plots indicating gating strategy to identify B cell subsets in mouse splenocytes.

Germinal center B cells were identified as B220+ CD95- CD38- IgD-. Plasmablasts were identified as B220+ CD138+ IgD-.

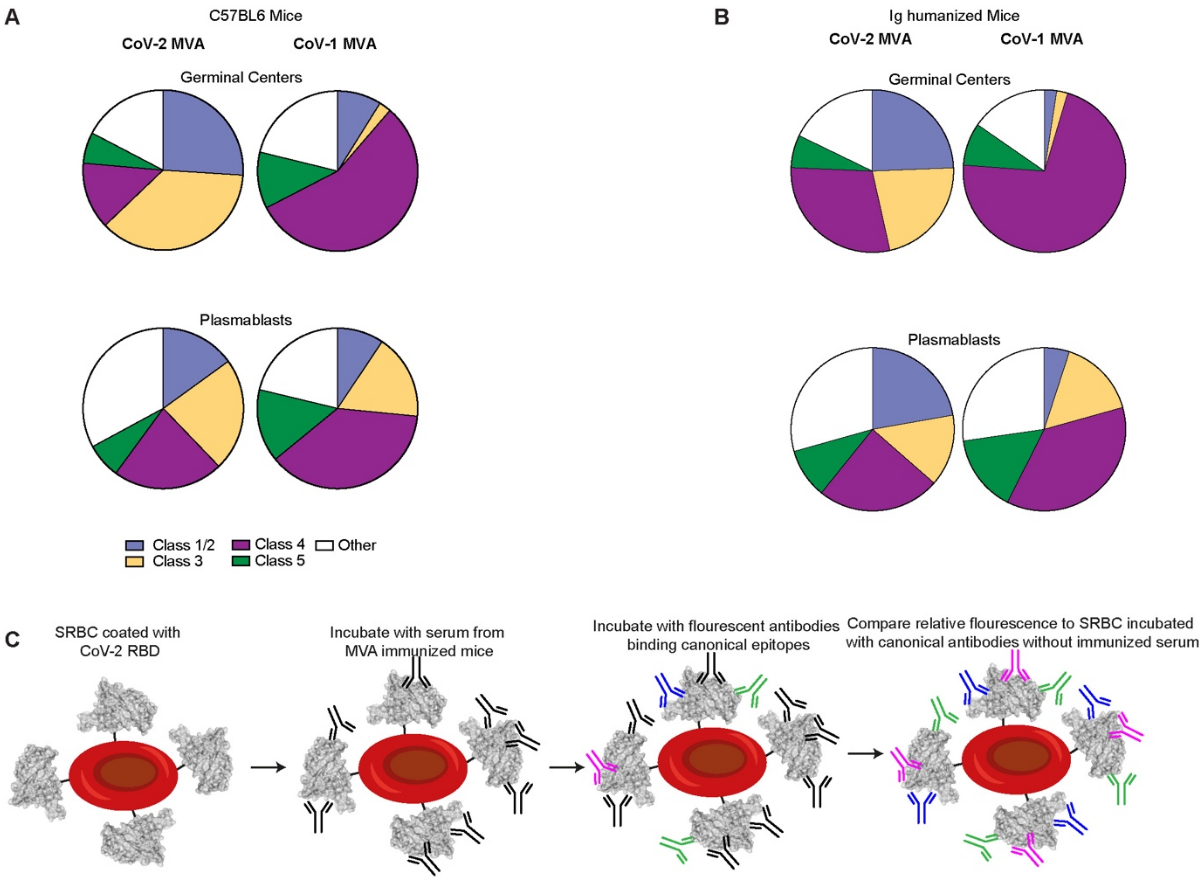

Figure S2. **Exploration of CoV-1 MVA and CoV-2 MVA as a primary immunization.** C57BL/6 mice were immunized with 2x10^{8} PFU IV of CoV-1, CoV-2 or control MVA on days 0 and 14 and the immune response examined on day 28.

A. Pie chart indicating the proportion of the total CoV-2 RBD binding response in the GC (top) or PB (bottom) compartment of C57BL/6 mice boosted with CoV-2 (left) or CoV-1 (right) MVA.

B. Pie chart indicating the proportion of the total CoV-2 RBD binding response in the GC (top) or PB (bottom) compartment of Ig-humanized mice boosted with CoV-2 (left) or CoV-1 (right) MVA.

C. Schematic illustration depicting the experimental approach utilized to assess the serum antibody response targeting the Class 1/2, Class 3, Class 4 or Class 5 epitopes of CoV-2. The approach involved conjugation of SRBC with CoV-2 RBD, followed by incubation with immunized mouse serum, and then incubation with fluorescent ACE2-Fc, S309, EY6A or S2H97 targeting the canonical Class 1/2, Class 3, Class 4 and Class 5 epitopes respectively. This allowed assessment of the relative reduction in MFI of the binding of these fluorescent canonical antibodies compared to a control sample when the fluorescent antibodies were added

without pre-incubation with immunized serum. The reduction in MFI compared to the no serum control was calculated as relative blocking %.

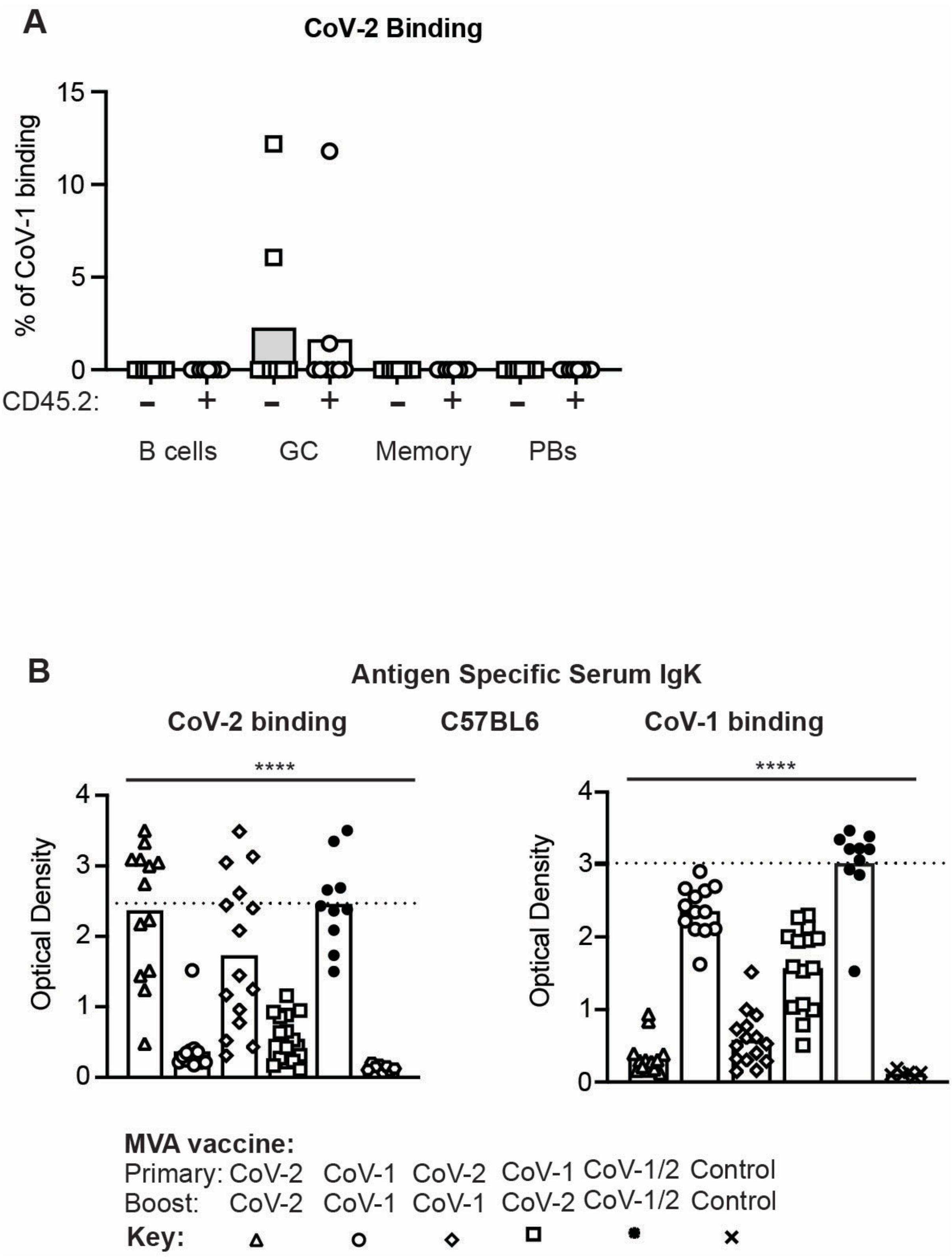

Figure S3. Role of cross-reactive B cells in the response of mice immunized with CoV-1 and CoV-2.

A. C57BL/6 (CD45.2) mice were immunized with 5ug/mouse IM of CoV-2 Spike mRNA vaccine BNT162b2 on day -14 and spleen cells harvested on day 0. These were mixed with an

equal number of spleen cells from naïve CD45.1 congenic mice and 2x106 cells transferred to RAG1-/- mice, and the recipients immunized with 2x108 PFU CoV-1 or control MVA. Splenic response was analyzed on day 14 after transfer. Graph presents % CoV-2 binding B cells amongst CoV-1 binding CD45.1 (squares) or CD45.2 (circles) GC, IgG1 memory or PB B cells.

B. C57BL/6 mice were immunized with 2x108 PFU of CoV-1 or CoV-2 or an equal mixture of CoV-1 and CoV-2 MVA on day 0. On day 14 mice were given a homologous or heterologous MVA immunization or the mixture again. Serum response was analyzed on day 28 to measure IgK response binding CoV-2 (left) or CoV-1 (right).

Data points represent individual mice. Columns denote arithmetic means. Statistical analysis by One-way ANOVA. ****p<0.0001.

Table S1: Epitope conservation across SARS-CoV-1 and SARS-CoV-2

A. Contact residues of the ACE2R and the S309, EY6A and S2H97 antibodies on the CoV-1 and CoV-2 RBD.
B. Pairwise comparison of the conservation of the contact residues of the ACE2R, S309, EY6A and S2H97 antibodies across the SARS-CoV-1 and CoV-2 RBD.
